# Influence of secondary neutrons induced by proton radiotherapy for cancer patients with implantable cardioverter defibrillators

**DOI:** 10.1186/1748-717X-7-10

**Published:** 2012-01-29

**Authors:** Takayuki Hashimoto, Tomonori Isobe, Haruko Hashii, Hiroaki Kumada, Hiroshi Tada, Toshiyuki Okumura, Koji Tsuboi, Takeji Sakae, Kazutaka Aonuma, Hideyuki Sakurai

**Affiliations:** 1 Department of Radiation Oncology, Graduate School of Comprehensive Human Sciences, University of Tsukuba, 1-1-1 Tennodai, Tsukuba, Ibaraki 305-8575, Japan; 2 Department of Cardiovascular Medicine, Graduate School of Comprehensive Human Sciences, University of Tsukuba, 1-1-1 Tennodai, Tsukuba, Ibaraki 305-8575, Japan

**Keywords:** Proton radiotherapy, Secondary neutrons, Implantable cardioverter defibrillator, Soft error, Monte-Carlo simulation

## Abstract

**Background:**

Although proton radiotherapy is a promising new approach for cancer patients, functional interference is a concern for patients with implantable cardioverter defibrillators (ICDs). The purpose of this study was to clarify the influence of secondary neutrons induced by proton radiotherapy on ICDs.

**Methods:**

The experimental set-up simulated proton radiotherapy for a patient with an ICD. Four new ICDs were placed 0.3 cm laterally and 3 cm distally outside the radiation field in order to evaluate the influence of secondary neutrons. The cumulative in-field radiation dose was 107 Gy over 10 sessions of irradiation with a dose rate of 2 Gy/min and a field size of 10 × 10 cm^2^. After each radiation fraction, interference with the ICD by the therapy was analyzed by an ICD programmer. The dose distributions of secondary neutrons were estimated by Monte-Carlo simulation.

**Results:**

The frequency of the power-on reset, the most serious soft error where the programmed pacing mode changes temporarily to a safety back-up mode, was 1 per approximately 50 Gy. The total number of soft errors logged in all devices was 29, which was a rate of 1 soft error per approximately 15 Gy. No permanent device malfunctions were detected. The calculated dose of secondary neutrons per 1 Gy proton dose in the phantom was approximately 1.3-8.9 mSv/Gy.

**Conclusions:**

With the present experimental settings, the probability was approximately 1 power-on reset per 50 Gy, which was below the dose level (60-80 Gy) generally used in proton radiotherapy. Further quantitative analysis in various settings is needed to establish guidelines regarding proton radiotherapy for cancer patients with ICDs.

## Background

Radiation therapy (RT) is a well-established modality for cancer treatment. It has been estimated that about 25% of all patients with cancer in Japan will require RT for cancer in their lifetime [1]. This percentage is approximately double in the United States and European countries. Since the projected increase in the elderly population of developed countries is the greatest in Japan, RT is expected to play an increasingly important role in Japan as well. Implantable cardioverter defibrillators (ICDs) are relatively large pacemakers that are also able to deliver a high voltage shock in case of a life-threatening ventricular tachycardia or ventricular fibrillation. They were first introduced in the early 1980s and have become more common in patients at high risk for sudden cardiac death. Random access memory (RAM), which is currently used in practically all cardiac pulse generators, is responsible for the high sensitivity of ICDs to ionizing radiation [2,3]. The literature reports that radiation therapy with high-dose ionizing radiation is associated with an increasing risk for adverse outcomes in patients with ICDs with malignant disease [4,5].

Proton radiotherapy is a new mode of radiation therapy that gives excellent dose distributions to the target. Recently, the number of proton facilities has been increasing worldwide. Direct proton beam irradiation on a cardiac pulse generator can permanently destroy electrical components, but this is seldom seen in the clinical setting. Secondary neutrons represent a major fraction of the radiation generated during proton radiotherapy from proton-induced nuclear reactions both in beamline components and in the patient's body, so secondary neutrons have been previously measured by various detectors or estimated by Monte-Carlo simulation using phantoms that are designed to represent human tissue [6-10]. As a large proportion of malignant tumors and selected benign diseases are treated with proton radiotherapy, hazards linked to the effect of secondary neutrons on ICDs may cause clinical problems in ICD-bearing patients. For such patients, it is still unclear whether the use of proton radiotherapy is safe, and this uncertainty reduces the number of tumor treatment possibilities. Therefore, it is important to establish the safety of delivering proton beams for patients with ICDs.

In the present study, ICDs in current clinical use were tested with proton beam irradiation in order to assess safety and ICD malfunction during proton beam therapy.

## Methods

### ICDs

Four new ICDs (Marquis DR 7274, Medtronic, Minneapolis, MN) were used in the present experimental study. These ICDs are indicated for ventricular anti-tachycardia pacing and ventricular defibrillation for automated treatment of life-threatening ventricular arrhythmias. The external shield of the ICD is titanium, and the ICD is 36 mL in volume (68.3 × 50.8 × 13.7 mm^3^) with a mass of 75 g. Parameters such as pacing mode and sensing threshold are programmed telemetrically, detected abnormalities are automatically recorded, and serious errors are recorded and alerted to with a sound. Prior to the experiments, all ICDs had not been previously exposed to radiation and were programmed to normal settings with detection and therapies set to "off" in order to avoid unexpected discharge.

### Types of errors observed in the ICDs

Malfunctions of the ICDs used in the present experiment were divided into four types, and are summarized in Table 1. Hard errors, the most serious type, cause a permanent stop in pulse generation. In the case of a hard error, recovery is mostly incomplete, and the device cannot be used reliably thereafter and must be replaced. A soft error is also known as single event upset (SEU), and soft errors were classified into three types. Power-on reset (POR), the most critical soft error, indicates accidental overwriting of important and protected device data that are essential for set-up of the pacing function and arrhythmia detection. In this condition, the pacing mode changes temporarily to the safety mode. Recovery may occur after the ICD is reprogrammed. A partial electrical reset (PER) and a POR both interfere with protected RAM data and cause the alarm to sound. However, PER does not create a change in the pacing mode or rate, because in the case of a PER the position of the overwritten data in the RAM does not overlap with the position in the RAM for pacing mode or arrhythmia detection settings. Minor errors cannot be detected by the programmer directly; they are revealed only by analysis of the device's data log.

**Table 1:** Type, description and total number of ICD errors caused by the secondary neutron from 107 Gy proton beam irradiation for each of four ICDs (Marquis DR 7274, Medtronic, Minneapolis, MN)

Type of error	Explanation	Change in frequency and pulse width	Alarm is sounded	ICD reprogramming	Number of sessions detected by the programmer	Number of errors revealed by analysis of the ICD data logs
Hard error	The ICDs stop generating pulses permanently.	Yes	No	Impossible	0	0
Soft error					13	29
Power-on reset	The ICDs present sudden complete failure and switch over to the safety backup mode.	Yes	Yes	Necessary	8	8
Partial electrical reset	The ICDs show no change in mode of pacing.	No	Yes	Unnecessary	5	7
Minor error	The ICDs show no change in mode of pacing.	No	No	Unnecessary	0	14

Abbreviation: ICD = implantable cardioverter defibrillator

After completion of each course of irradiation, ICD parameters such as pacing mode and pulse rate were analyzed by the programmer in-house. Data logs from the ICDs were sent to the manufacturer for more detailed analysis.

### Proton radiotherapy equipment

Proton beams of 200 MeV (a spread in energy of σ = 0.8 MeV) generated by a synchrotron with a linear accelerator (PROBEAT; Hitachi, Tokyo, Japan) at the Proton Medical Research Center (PMRC), University of Tsukuba were used in the present study.

**Figure 1:**
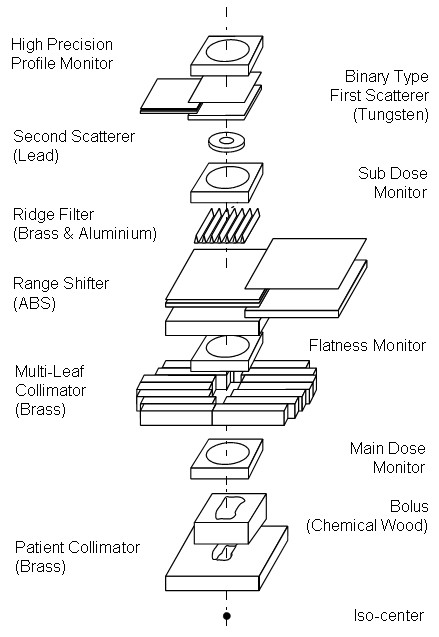
**Schematic of the proton beam delivery system**.

The proton radiotherapy system in the treatment room consisted of an isocentrically rotating gantry equipped with a biplane digital radiography unit and a treatment couch. The proton irradiation method applied at PMRC is the passive scattering method. Proton beams were spread out and shaped with a ridge filter, double-scatterer, multileaf collimators, and a custom-made bolus that covered the target volume (Figure 1). The patient bolus, which is normally mounted at the shield ring, was not considered in this work.

### Experimental settings

**Figure 2:**
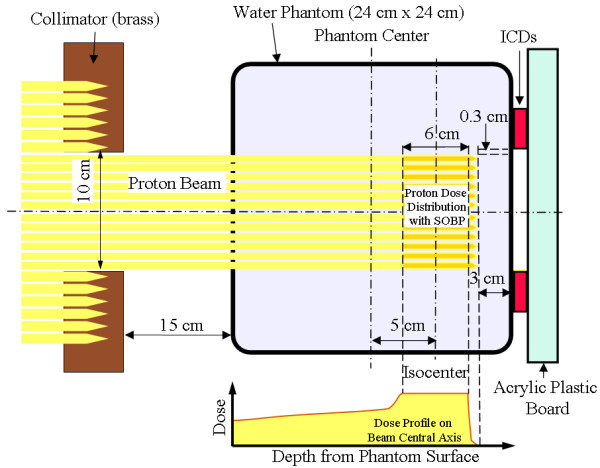
**View of the experimental set-up of proton irradiation to the phantom with implantable cardioverter defibrillators (ICDs) on the central axis of the proton beam**. Four ICDs were placed on the contralateral side of the phantom. Proton beams entered the water phantom laterally, and an acrylic plastic board was set behind the ICDs to provide backscatter conditions. Calculated spread-out Bragg peak (SOBP) curves in water and experimental values are included.

Figure 2 shows the experimental set-up of proton irradiation to the phantom with ICDs. As predicted from previous clinical data, the frequency of soft errors was very low [11].

Therefore, four ICDs were set on the back surfaces of a water phantom (external dimension: 24 × 24 × 24 cm^3^) in order to raise the probability of the occurrence of soft errors. Proton beams entered the lateral side of the phantom, and the distal end of the spread-out Bragg peak (SOBP) was 3 cm in front of and 0.3 cm inside of the ICDs. In other words, the devices were placed outside the field of direct and secondary proton beams in order to focus on and observe the influence of secondary neutrons on the ICDs. Moreover, in order to provide backscatter conditions, a 20-mm-thick acrylic plastic board was placed behind the devices [12]. The dose rate was 2 Gy/min and the field size was 10 × 10 cm^2^. The SOBP was 6 cm in length. A total of 107 Gy was delivered in 10 sessions of irradiation at the SOBP center, and ranged from 2 to 20 Gy in one session. The ICDs were monitored during proton beam irradiation in order to detect the alarm sound that is produced by the device to notify the occurrence of serious soft errors.

### Dose distributions of secondary neutrons

Neutrons were classified according to their kinetic energy as thermal (*E *< 0.5 eV), epithermal (0.5 eV<*E *< 10 keV) or fast (*E *> 10 keV). Dose distributions of total secondary neutrons were estimated by numerical simulation with the Monte-Carlo method utilizing the particle and heavy ion transport code system (PHITS) developed by the Japan Atomic Energy Agency: JAEA, Japan. Details of the distribution of secondary neutrons have been previously described [13,14].

## Results

Table 1 shows the numbers and types of errors observed in the present experiment. In POR, the ICDs presented sudden complete failures, and reverted back to initial factory-programmed settings. Additionally, the pacing rate changed to 65 beats/min, which was not a manually programmable rate. PERs or minor errors did not influence the function of the ICDs after completion of treatment. After a POR, the ICDs functioned normally after reprogramming.

Telemetry analysis by the programmer in-house after completion of each course of irradiation revealed soft errors in 13 of 40 courses of irradiation (Table 1). According to detailed analysis of data logs in the ICDs, multiple errors occurred in 4 courses during 1 course of irradiation (POR + PER in 2, PER + a minor error in 1, 3 minor errors in 1) (Table 1). The frequency of POR was approximately 1 per 50 Gy. The total number of critical and minor soft errors was 29, for a rate of approximately 1 per 15 Gy. The timing of the occurrence of errors was unpredictable and random from the start to the end of the irradiation, and the frequency of the errors did not increase with accumulated irradiation dose. Hard errors were not observed in this experimental study, and the ICDs in the initial factory-programmed settings retained sensitivity of detecting arrhythmia and continued to generate pulses.

**Figure 3:**
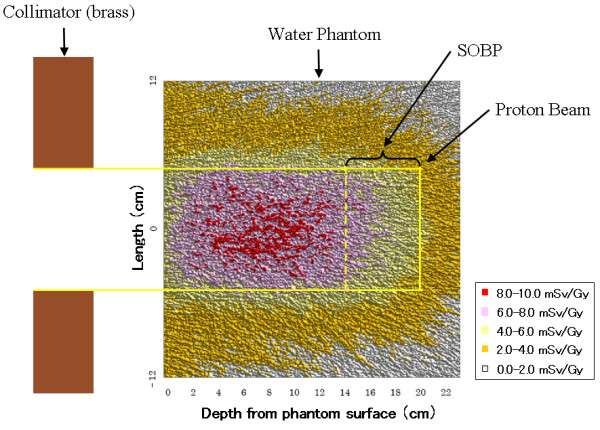
**Results of predicted dose due to secondary neutrons in the phantom determined by the Monte-Carlo calculation**.

**Figure 4:**
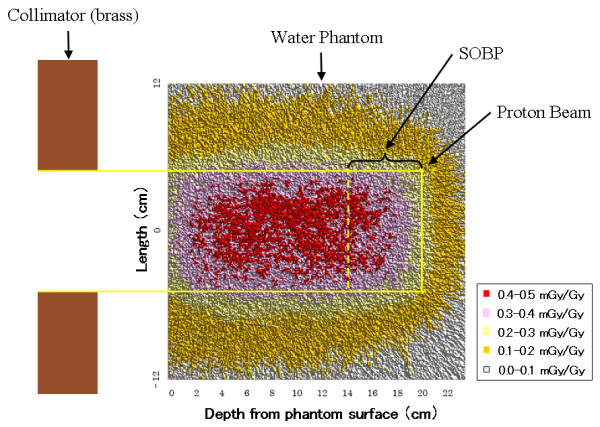
**Results of predicted dose due to gamma-rays in the phantom determined by the Monte-Carlo calculation**.

Figures 3 and 4 show the results of predicted dose as determined by the Monte-Carlo calculation. The estimated dose of secondary neutrons per 1 Gy proton dose was approximately 2.7 mSv/Gy for the ICDs, and was approximately 1.3-8.9 mSv/Gy in the phantom. The gamma-ray dose per 1 Gy proton dose in the phantom was approximately 0.11-0.45 mGy/Gy, and the ratio of the gamma-ray dose at the beam central axis was about 5% of the total secondary radiation dose.

## Discussion

Recently, some authors have reported a damaging effect of therapeutic radiation on cardiac pulse generators [4,5,15]. It is well known that ionizing radiation can interfere with RAM in cardiac pulse generators. Recommendations on the management of patients with cardiac pulse generators undergoing radiotherapy are based on limited studies mostly involving pacemakers [16,17]. The American Association of Physicists in Medicine (AAPM) has published a review of contemporary cardiac pacemaker failure due to radiation damage [3]. The AAPM task group suggested that pacemakers should not be placed in the direct therapy beam, and the maximum dose absorbed to the pacemaker should be below 2 Gy. In the present study, the gamma-ray dose in the phantom was only about 0.11-0.45 mGy/Gy, which was far below the dose of 2 Gy. Therefore, secondary gamma-rays are considered to have no important effect on cardiac pulse generators. However, the AAPM reports were based only on experience with pacemakers. Kapa et al. reported that components of ICDs are more vulnerable than pacemakers to the ionizing effects of radiation [4]. Even when devices are kept out of the direct radiation field during external-beam radiotherapy using high-energy photons (e.g., ≥ 10 MV), device malfunction can occur at minimal in-field dose levels. Lau et al. reported a case of electrical reset of an ICD by scattered irradiation from radiotherapy for a patient with prostate cancer [18]. The device alarm went off during external-beam radiotherapy to the pelvis using 23 MV photons. Secondary neutrons could have been the cause of device malfunction in that report. Raitt et al. reported the influence of fast neutron radiotherapy on a pacemaker lying outside the treatment field [19]. The very low estimated dose of 0.9 Gy received by the pacemaker demonstrated the extreme sensitivity of integrated circuits. Although fast neutron radiotherapy is not commonly used because of its unacceptably high incidence of late morbidity, questions have been raised concerning secondary neutrons produced by external-beam radiotherapy using high-energy photons or by particle therapy.

In proton radiotherapy using the passive scattering irradiation method, proton beams generate secondary neutrons by the reaction with the collimator and several other scattering components [6,20]. On the other hand, Schneider et al. reported that the spot scanning technique showed a dose advantage at a beam line of at least 10 times over the scatter foil technique [21]. In the healthy tissues of their patient (in the non-treated volume), the dose coming from neutrons was approximately 0.002-0.004 Sv per treatment Gy. These contributions to the integral dose from neutrons are very low, so they concluded that the dose deposited by secondary neutrons during proton radiotherapy using the spot scanning technique can be neglected in the treatment region. However, proton beams also generate secondary neutrons and photons by the reaction with several elements that form human body tissue. The internal incidental dose around the center at deeper situated regions accounted for about 60% to 80% of the total incidental dose [14]. Therefore, in proton radiotherapy, it is impossible to completely eliminate the influence of secondary neutrons, even if shielding of external neutrons or active scanning method are used.

Morávek et al. reported that the neutron contribution to the dose behind the peak maximum was at least 3 orders smaller than the total dose at the peak maximum [7]. Furthermore, it decreased exponentially with the distance to the peak maximum. Therefore, they concluded that its influence on the dose distribution is marginal. However, it should be pointed out that their work refers only to the physical dose and does not take into account the influence on ICDs. To date, there have been few studies of the interactions between ICDs and secondary neutrons from proton radiotherapy. We previously reported that changes in heart rate occurred in 2 of 8 cancer patients with pacemakers who received proton radiotherapy in our facility [11]. Potential hazards of proton beam irradiation for patients using the new generation of cardiac pulse generators with digital circuitry are not yet well known. However, in order to keep open as many options for cancer treatment as possible, the judgment of contraindication of proton radiotherapy for cancer patients with cardiac pulse generators should be made carefully. To assess the safety and influence of proton radiotherapy on ICDs, the present experimental study was conducted. To our knowledge, this is the first report on the influence of secondary neutrons generated by proton radiotherapy on ICDs. Devices failed at the rate of approximately 1 failure per 15 Gy, which is well below the dose level (60-80 Gy) generally used in proton radiotherapy. The probability of a soft error caused by secondary neutrons induced by proton radiotherapy on ICDs is very small, but it is an inevitable and unpredictable phenomenon. Rodriguez et al. reported on radiation-induced effects in multi-programmable pacemakers and ICDs [22]. Pacemaker malfunction induced by ionizing-radiation exposure is unpredictable, because these effects can occur in multiple locations in complementary metal-oxide semiconductor (CMOS) and do so at random. Therefore, errors could potentially be observed even at the minimal delivered dose, and relocation of the device out of the radiation field is not enough to prevent the occurrence of soft errors. Minor errors which cannot be detected by the programmer directly were often observed, suggesting that the device could be damaged by secondary neutrons even if the device malfunction is not apparent. Hard errors of the ICDs were not observed in the present study, and the devices in their initially programmed settings always kept their sensitivity and generating pulses. Further investigation is needed to clarify whether the total cumulative radiation dose to the device results in an increased likelihood of soft errors, and how much the ratio of fast or thermal neutrons contributes to the causes of soft error.

The experimental findings of the present study have resulted in the recommendation in our department for the use of this new cancer treatment modality for patients with cardiac pulse generators. It is essential that patients be monitored carefully during the course of treatment and that the pacing mode and rate be monitored after completion of irradiation in accordance with the degree of dependence on the cardiac pulse generators. After the completion of each radiation session, the device should be interrogated in order to find all abnormalities. If possible, pre-treatment involving experimental evaluation for each device is preferable, because the effects of secondary neutrons varied between the devices.

It should be pointed out that the measurements in the present study were performed under a limited set of standard physical conditions. To clarify how the delivered dose and position of the device influence the function of ICDs, further corrections are necessary for the standard geometry in which patients with cancer are irradiated in the clinical setting. In addition, the same experimental irradiation should be tested in other facilities with either equipment for particle therapy or with linear accelerators with a capacity of over 10 MV X-ray output. Furthermore, therapeutic guidelines concerning the safe use of proton radiotherapy for patients bearing cardiac pulse generators are needed.

## Conclusions

Soft errors caused by secondary neutrons induced by proton radiotherapy on ICDs are rare, but are an inevitable and unpredictable phenomenon. Although the present study was performed under a limited set of clinical conditions, the calculated dose of secondary neutrons per 1 Gy proton dose was approximately 2.7 mSv/Gy to the ICDs, and was approximately 1.3-8.9 mSv/Gy to the phantom. Approximately 1 power-on reset occurred per 50 Gy, which was below the dose level (60-80 Gy) generally used in proton radiotherapy. Further quantitative analysis in various settings is needed to establish guidelines regarding proton radiotherapy for cancer patients with ICDs.

## Competing interests

We have no personal or financial conflicts of interest and have not entered into any agreement that could interfere with our access to the data in the research, upon our ability to analyze data independently, to prepare manuscripts, or to publish them.

## Authors' contributions

TH and TI developed the ideas for these studies, performed much of the work, and drafted the manuscript. HH participated in the acquisition and analysis of data. HK and KT revised the manuscript and provided important intellectual content. TS and TO participated in interpretation of the data and revision of the manuscript. HT and KA participated in the study design and revision of the manuscript. HS participated in the study design, interpretation of the data, and was responsible for final approval of the manuscript. All authors have read and approved the final manuscript.
